# Presumptive Progressive Multifocal Encephalopathy in an Immunocompetent Patient: A Rare Case Report

**DOI:** 10.7759/cureus.46668

**Published:** 2023-10-08

**Authors:** Minh Ngo, Ngan Tang, Quang Le

**Affiliations:** 1 Neurology, University of Missouri, Columbia, USA; 2 Neurology, Ho Chi Minh City Stroke Association, Ho Chi Minh, VNM; 3 Hospital Medicine, University of Missouri School of Medicine, Columbia, USA

**Keywords:** drug abuse, immunocompetent individuals, progressive multifocal leukoencephalopathy (pml), jcv encephalitis, john cunningham virus

## Abstract

Progressive multifocal encephalopathy (PML) is a rare brain infection caused by the John Cunningham virus (JCV), primarily affecting immunocompromised individuals. This case report presents a unique occurrence of PML in an immunocompetent young man with a history of substance abuse. The patient exhibited progressive neurological symptoms, including weakness and sensory deficits, prompting diagnostic evaluation. Brain imaging and laboratory tests revealed evidence of PML, supported by a positive JCV antibody. Notably, HIV testing was negative. While PML is typically associated with immunosuppression, this case raises questions about potential connections between substance abuse and viral reactivation. The patient received treatment with intravenous methylprednisolone and underwent rehabilitation, emphasizing the challenging nature of PML management. This case highlights the importance of considering PML as a differential diagnosis, even in immunocompetent individuals, and underscores the need for further research into its rare presentations and associated risk factors.

## Introduction

Progressive multifocal encephalopathy (PML) is a potentially devastating brain infection caused by the John Cunningham virus (JCV). This insidious disease can lead to severe neurological disability or even death. While PML is typically associated with individuals who have compromised immune systems, such as those with HIV/AIDS, it is extremely rare in immunocompetent individuals. The exact mechanisms behind how JCV causes PML in such individuals are not fully understood. The condition poses a significant clinical challenge due to the absence of a definitive cure. Currently, the primary approach to managing PML involves providing supportive care to alleviate symptoms and address complications when they arise. In this case study, we present a particularly rare occurrence of PML in a young and otherwise immunocompetent man, shedding light on the complex nature of this condition along with its potential therapy.

## Case presentation

A 32-year-old, right-handed male came to the emergency room with progressive left-sided weakness for three days. Other symptoms included slurred speech and paresthesia, more prominently in the left upper extremity. He denied a rash, fever, headache, neck pain, dizziness, or any visual changes. Past medical history was significant for polysubstance and tobacco abuse. He admitted using IV methamphetamine and smoked cocaine five days prior to this admission. His vitals remained stable. The clinical exam was remarkable for weakness with muscle strength of 3/5 and superficial sensory deficits on the left side. There was no change in bowel movement or urination. Routine blood work with a complete blood count, metabolic panel, inflammatory markers, and hypercoagulable panel remained negative (Table 1).

**Table 1:** Routine blood work WBC: white blood count, CRP: C-reactive protein, ESR: erythrocyte sedimentation rate, TSH: thyroid-stimulating hormone

Test	Result	Ref range & Unit
Blood Count		
WBC	4.77	4.0-10.8 x 10^3/uL
Hemoglobin	15.3	13-18 g/dL
Platelet	167	150-350 x 10^3/uL
HbA1c	5.5	<5.7 %
Vitamin B12	584	193-986 pg/mL
TSH	0.661	0.358-3.740 uIU/mL
Inflammatory Markers		
CRP	4.38	<0.8 mg/dL
ESR	33	0-14 mm/hour

**Figure 1:**
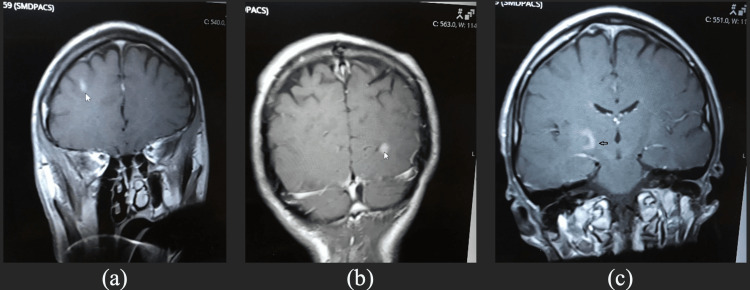
MRI of the brain with T2-weighted images (a) Hyperintensive lesion in the right frontal lobe. (b) Left occipital lobe lesion. (c) A thin rim enhancement in the medial right of the internal capsule lesion.

The urine drug screen was positive for amphetamine, cocaine, and marijuana. Head CT and CT angiography of the head and neck revealed no significant findings. However, an MRI of the brain showed abnormally enhanced bi-hemispheric lesions mostly related to white matter favoring the inflammatory process (Figure 1).

Cervical, thoracic, and lumbar spine MRI also demonstrated tiny patchy areas of the lower thoracic spinal cord concerning demyelinating disease. We empirically administered a short course of intravenous methylprednisolone for five days and discontinued it after the multiple sclerosis (MS) panel including myelin oligodendrocyte glycoprotein and aquaporin-4 immunoglobulin G (IgG) antibodies came back negative (Table 2). However, the serum JCV antibody was positive. Subsequent 4th-generation HIV testing was negative (Table 2). Unfortunately, the patient declined a cerebral spinal fluid (CSF) study. He was managed conservatively with a course of inpatient rehabilitation and discharged with a close plan for outpatient follow-up. 

**Table 2:** Autoimmune and infectious disease panel Ab: antibody, MOG: myelin oligodendrocyte glycoprotein, ANA: antinuclear antibody, HIV: human immunodeficiency virus, DRVVT: dilute Russell viper venom time, HBV: hepatitis B virus, HCV: hepatitis C virus, HAV: Hepatitis A virus

Test	Result
Myeloperoxidase Ab	Negative
Proteinase-3 Ab	Negative
Aquaporin-4 IgG	Negative
MOG Ab	Negative
Cardiolipin Ab (IgG/IgA/IgM)	Negative
Lupus anticoagulant	Negative
ANA	Negative
DRVVT screen	Negative
Hepatitis panel (HAV, HBV, HCV)	Negative
HIV 1/2 AB + HIV1 P24 AG	Negative
Lyme IgG/IgM	Negative
Syphilis IgG/IgM	Negative
JCV Ab	Positive

## Discussion

JCV is common and harmless in immunocompetent individuals and remains latent; about 80% of the human population is JCV antibody-positive prior to adulthood [1-3]. It persists in kidney, bone marrow, and spleen tissues until it is reactivated to reach the brain via B-cells under immunosuppressed conditions, causing PML [4]. However, several investigations have surprisingly found the JCV genome not only in the brain tissue of PML patients but also in the brain tissue of immunocompetent individuals [2,3]. This may lead to a few unexplained and exceedingly rare cases of PML in patients without immunodeficiency [5]. PML’s clinical features include lateral or unilateral progressive weakness, paresthesia, visual loss, or mental status change [5]. MRI of the brain often demonstrates demyelinating processes involving hemispheric white matter in multiple brain lobes [6]. Brain biopsy helps make a definitive diagnosis of PML. In the absence of a biopsy, a diagnosis of PML can be confirmed by brain imaging or by the appearance of the JCV genome in the polymerase chain reaction (PCR) of the cerebral spinal fluid (CSF) [5]. MS is one of the most important differential diagnoses, especially given similar MRI features. In this patient, a negative MS antibody panel, along with other characteristics of age, sex, and acute presentation, make MS a less favorable diagnosis. On the other hand, neurologic symptoms, along with MRI lesions and a positive JCV antibody, can make a presumptive PML diagnosis acceptable, though we are not able to collect a CSF study [7]. The patient denied any history of cancer and did not take any medications. He carries a high risk for HIV transmission; however, HIV testing came back negative. Despite the high sensitivity of the fourth-generation testing, it is still possible that the result may be falsely negative within the window period. Our case report may also have some limitations; there might be an extra need to rule out other potential immune disorders, such as common variable immunodeficiency, which may also reactivate JCV. In this patient, another possible explanation is that the history of polysubstance abuse can lead to transient dysfunction of the immune system, resulting in JCV reactivation. In fact, there may be a link between marijuana use and immune suppression, thus increasing the risk of viral infections [8]. PML often causes fatality in most cases, immunocompetent patients may have a higher chance of survival; possibly linked to methylprednisolone use [9]. Overall, due to the rarity of the disease, no effective antiviral treatment for PML has been identified yet. An in-vitro study suggests mefloquine as a potential agent; however, testing the drug in vivo poses a great challenge due to the lack of testing animals with PML [10].

## Conclusions

PML caused by reactivated JCV infection is an uncommon occurrence, especially in healthy individuals. There is a possible link with substance abuse. Diagnosis and treatment approaches remain challenging with high mortality rates. The prognosis may be more favorable in an immunocompetent population.
